# Yi Shen Juan Bi Pill alleviates bone destruction in inflammatory arthritis under postmenopausal conditions by regulating ephrinB2 signaling

**DOI:** 10.3389/fphar.2022.1010640

**Published:** 2022-09-30

**Authors:** Huihui Xu, Li Tao, Jinfeng Cao, Peng Zhang, Hui Zeng, Hongyan Zhao

**Affiliations:** ^1^ Department of Bone & Joint Surgery and National & Local Joint Engineering Research Center of Orthopaedic Biomaterials, Peking University Shenzhen Hospital, Shenzhen, China; ^2^ Center for Translational Medicine Research and Development, Shenzhen Institutes of Advanced Technology, Chinese Academy of Sciences, Shenzhen, China; ^3^ Beijing Key Laboratory of Research of Chinese Medicine on Prevention and Treatment for Major Diseases, Experimental Research Center, China Academy of Chinese Medical Science, Beijing, China

**Keywords:** collagen-induced arthritis, ovariectomy, bone destruction, Yi Shen Juan Bi Pill, ephrinB2

## Abstract

Yi Shen Juan Bi Pill (YSJB) is a traditional Chinese medicine (TCM) formulation that has a therapeutic effect upon rheumatoid arthritis (RA), but how YSJB affects bone destruction in arthritis under postmenopausal conditions is not known. We evaluated the therapeutic role of YSJB in bone destruction in postmenopausal arthritis, We used collagen-induced arthritis (CIA) rats who had been ovariectomized (OVX) as models and explored the possible mechanism from the synovium and bone marrow (BM). Arthritis was generated after ovariectomy or sham surgery for 12 weeks. After 14 days of primary immunization, rats were administered YSJB or estradiol valerate (EV) for 28 days. YSJB could prevent bone destruction in the inflamed joints of rats in the OVX + CIA group. CIA promoted osteoclast differentiation significantly in the synovial membrane according to tartrate resistant acid phosphatase (TRACP) staining, and OVX tended to aggravate the inflammatory reaction of CIA rats according to hematoxylin-and-eosin staining. Immunohistochemistry revealed that the synovium did not have significant changes in erythropoietin-producing hepatocellular interactor (ephrin)B2 or erythropoietin-producing hepatocellular (eph) B4 expression after YSJB treatment, but YSJB treatment reduced nuclear factor of activated T cells (NFATc)1 expression. The BM of rats in the OVX + CIA exhibited remarkable increases in the number of osteoclasts and NFATc1 expression, as well as significantly reduced expression of ephrinB2 and ephB4 compared with the CIA group and sham group. YSJB treatment reduced NFATc1 expression significantly but also increased ephrinB2 expression in the BM markedly. These data suggest that YSJB exhibit a bone-protective effect, it may be a promising therapeutic strategy for alleviating bone destruction in arthritis under postmenopausal conditions, and one of the mechanisms is associated with the modulation of ephrinB2 signaling.

## Introduction

Rheumatoid arthritis (RA) is a systemic autoimmune disease characterized by inflammatory synovitis, cartilage erosion, and bone destruction. RA prevalence worldwide is ∼1% (van der Woude and van der Helm-van Mil, 2018). RA prevalence in women is higher than that in men, and the former experience increased disability and greater functional decline than men (Kvien et al., 2006; Wallenius et al., 2009). Changes in gonadal hormones may participate in these differential immune responses in RA (Pennell et al., 2012). For instance, estrogen deficiency (which is attributed to aging and menopause) is related to RA progression (Shah et al., 2020). Menopause has a significant impact on worsening progression of functional decline in women with RA, which is not observed in premenopausal women compared with postmenopausal women (Mollard et al., 2018). Postmenopausal RA patients are susceptible to considerable bone damage and disability (Sammaritano, 2012). Therefore, discovering new targets for RA treatment after menopause are crucial.

In traditional Chinese medicine (TCM) theory, kidney deficiency syndrome is one of the most common syndromes of RA and corresponds (at least in part) to low levels of gonadal hormones. Yi Shen Juan Bi Pill (YSJB) is a TCM formulation which is used to treat RA patients with kidney deficiency syndrome. Previously, we demonstrated that YSJB protected against collagen-induced arthritis (CIA) in rats with a castration-induced kidney-deficiency pattern (Zhao et al., 2012). Moreover, we showed that YSJB ameliorated systemic bone loss/destruction in CIA rats, which affected the activation of osteoclasts and regulated osteoclast-mediated bone resorption by inhibiting expression of receptor activator of nuclear factor-kappa B (RANK), nuclear factor of activated T cells (NFATc)1, and c-fos (Zhao et al., 2018; Xia et al., 2021).

Bone destruction and general bone loss in RA are considered to be related to abnormal activation of osteoclasts (Kim et al., 2020; Liu et al., 2020). Interactions between osteoclasts and osteoblasts by erythropoietin-producing hepatocellular interactor (ephrin) ligands and erythropoietin-producing hepatocellular (eph) receptors have crucial roles in maintaining bone homeostasis (Tazaki et al., 2018), and they also participate in RA pathogenesis (Romanovsky, 2006). EphrinB2 is expressed on the membrane of mature osteoclasts. Interestingly, the osteoblast membrane expresses ephrinB2 and ephB4 simultaneously (Matsuo, 2010). The bidirectional signaling between osteoclastic ephrinB2 and osteoblastic ephB4 suppresses the bone resorption of osteoclasts and enhances the bone formation of osteoblasts (Zhao et al., 2006). Reverse signaling through ephrinB2 can inhibit NFATc1 transcription and thereby suppress osteoclast activity (Matsuo and Otaki, 2012). Enhancing ephrinB2–ephB4 signaling can inhibit osteoclastogenesis and prevent bone loss in ovariectomized (OVX) rats (Zhang et al., 2022).

Studies have found that inflammatory infiltrates are present in the deep part of the bone marrow (BM), far from the synovium–BM junction. Data from animal experiments have shown that the cortical-bone canaliculi connecting BM and synovium increase, which may help osteoclastic precursor cells migrate directly from the BM to the synovium, thus stimulating the “extraarticular” pathologic process of RA centered on the BM. According to the TCM theory “kidney governs bone and generates marrow” in many clinical and experimental studies, kidney-tonifying TCM formulations can inhibit bone absorption or promote bone formation.

We explored how YSJB influences bone destruction in arthritis under postmenopausal conditions with modulation of ephrinB2–ephB4 signaling from the synovium and BM.

## Materials and methods

### Ethical approval of the study protocol

The experimental protocol was approved (2016-030) by the Institute of Basic Theory of Traditional Chinese Medicine within the China Academy of Chinese Medical Sciences (Beijing, China).

### Animals

Seventy female adult Sprague‒Dawley rats (10 weeks) were purchased from the National Institutes for Food and Drug Control (animal license number: SCXK (Beijing) 2014-0013). Rats were kept in plastic cages (545 × 395 × 200 mm) with a maximum of five animals per cage under specific pathogen-free conditions in the Experimental Animal Center of the Institute of Basic Theory of Traditional Chinese Medicine [Experimental Animal Center license number: SYXK (Beijing) 2016-0021]. They were allowed to adapt to their environment for 7 days before experimentation initiation. Rats were housed in a room at 22°C ± 1°C with 45%–65% humidity under a 12-h light–dark cycle. They were provided with a normal chow diet and water *ad libitum*. The bodyweight of rats, water intake, and food consumption were assessed every week.

### Drugs

YSJB was provided by Nantong Liangchun Hospital of Traditional Chinese Medicine (Nantong, Jiangsu, China). Estradiol valerate (EV) was obtained from Bayer Delpharm Lille (Lys-lez-Lannoy, France).

### Ovariectomy

Rats were divided randomly into a sham group and OVX group by body weight. Rats were anesthetized using pentobarbital sodium (45 mg/kg, i.p.) and bilateral ovaries were removed from rats in the OVX group. Through flank incisions, only the adipose tissue near the ovaries was cut in rats in the sham group. Regrettably, two rats died during ovariectomy. After 11 weeks, OVX rats were subjected to collection of vaginal secretions (from which atrophic patterns were noted upon creation of vaginal smears).

### Collagen-induced arthritis induction

Twelve weeks after ovariectomy, an emulsion for primary injection was prepared, as reported previously (Xiao et al., 2009). Briefly, bovine type-II collagen (Chondrex, Woodinville, WA, United States) was emulsified with an equal amount of incomplete Freund’s adjuvant (Chondrex, Woodinville, WA, United States). Then, the emulsion (100 μg) was injected subcutaneously at the base of the tail of each rat. After 1 week, a secondary booster dose of 100 μg was given as the same preparation. The sham group received a subcutaneous injection of physiologic saline at the base of the tail. Arthritis severity was expressed as the arthritic index from 0 to four according to the following scale: 0, no signs of disease; 1, detectable arthritis with erythema in at least some digits; 2, significant redness and swelling; 3, severe redness and swelling from joint to digit; 4, maximal swelling with arthrokleisis. The maximum arthritic index score per rat was 8 (4 points × 2 hind paws).

### Experimental grouping

Fifteen days after primary immunization or the sham procedure, rats were divided randomly into five groups by the arthritic index score: sham (*n* = 12); CIA (*n* = 15); OVX + CIA (*n* = 13); OVX + CIA + EV (*n* = 14); OVX + CIA + YSJB (*n* = 14).

Rats in the OVX + CIA + YSJB group and OVX + CIA + EV group were administered 1.29 g/kg d and 0.11 mg/kg d, respectively, of the drug *via* the oral route. The other groups were administered an equal volume of pure water (1 ml/100 g).

### Histology

Rats were killed under anesthesia by cervical dislocation 4 weeks after drug administration. The right ankle and knee joints were dissected and fixed immediately in formalin for 7 days. The right joints were decalcified in 12.5% EDTA and embedded in paraffin. Tissue sections were stained with hematoxylin and eosin. The infiltration of cells into synovial tissue, cartilage, and bone damage was scored on a scale of 0–3 (0: absent; 1: weak; 2: moderate; 3: severe).

### Micro-computed tomography

The left hind paws and ankle joints were imaged and reconstructed into a three-dimensional (3D) structure using a micro-CT system (Skyscan 1174; Bruker, Billerica, MA, United States). The bone volume (BV) and bone surface (BS) of tarsal bones were analyzed to evaluate microstructural changes in bone. The BS/BV ratio was calculated to evaluate the surface density and focal erosion of periarticular bone.

### Tartrate resistant acid phosphatase staining

Sections of ankle joints were subjected to TRACP staining to identify osteoclastogenesis according to the instructions of a TRACP staining kit (MilliporeSigma, Burlington, MA, United States). TRACP^+^ multinucleated cells containing ≥3 nuclei were counted as osteoclasts. Specimens were evaluated by Qwin™ (Leica Microsystems, Wetzlar, Germany).

### Immunohistochemistry

EphrinB2, ephB4, and NFATc1 (Abcam, Cambridge, UK) were localized in ankle joints according to manufacturer’s instructions. Paraffin sections were dewaxed using routine methods and treated overnight with primary antibodies against ephrinB2, ephB4, and NFATc1 for rats at 4°C. Then, specimens were incubated with poly-horseradish peroxidase anti-rabbit immunoglobulin (Ig)G for 10 min at room temperature, stained with 3,3-diaminobenzidine, and counterstained with hematoxylin. As a control, rabbit IgG isotype (1:100 dilution) was used instead of primary antibodies. Specimens were analyzed, and positive cells were counted using Qwin™ (Leica Microsystems).

### Statistical analyses

Data were analyzed using SPSS 20.0 (IBM, Armonk, NY, United States). Data are the mean ± SD. Results were compared using one-way ANOVA. *p* < 0.05 was considered significant.

## Results

### YSJB reduces the arthritis index scores in OVX + CIA rats to prevent arthritis progression

**FIGURE 1:**
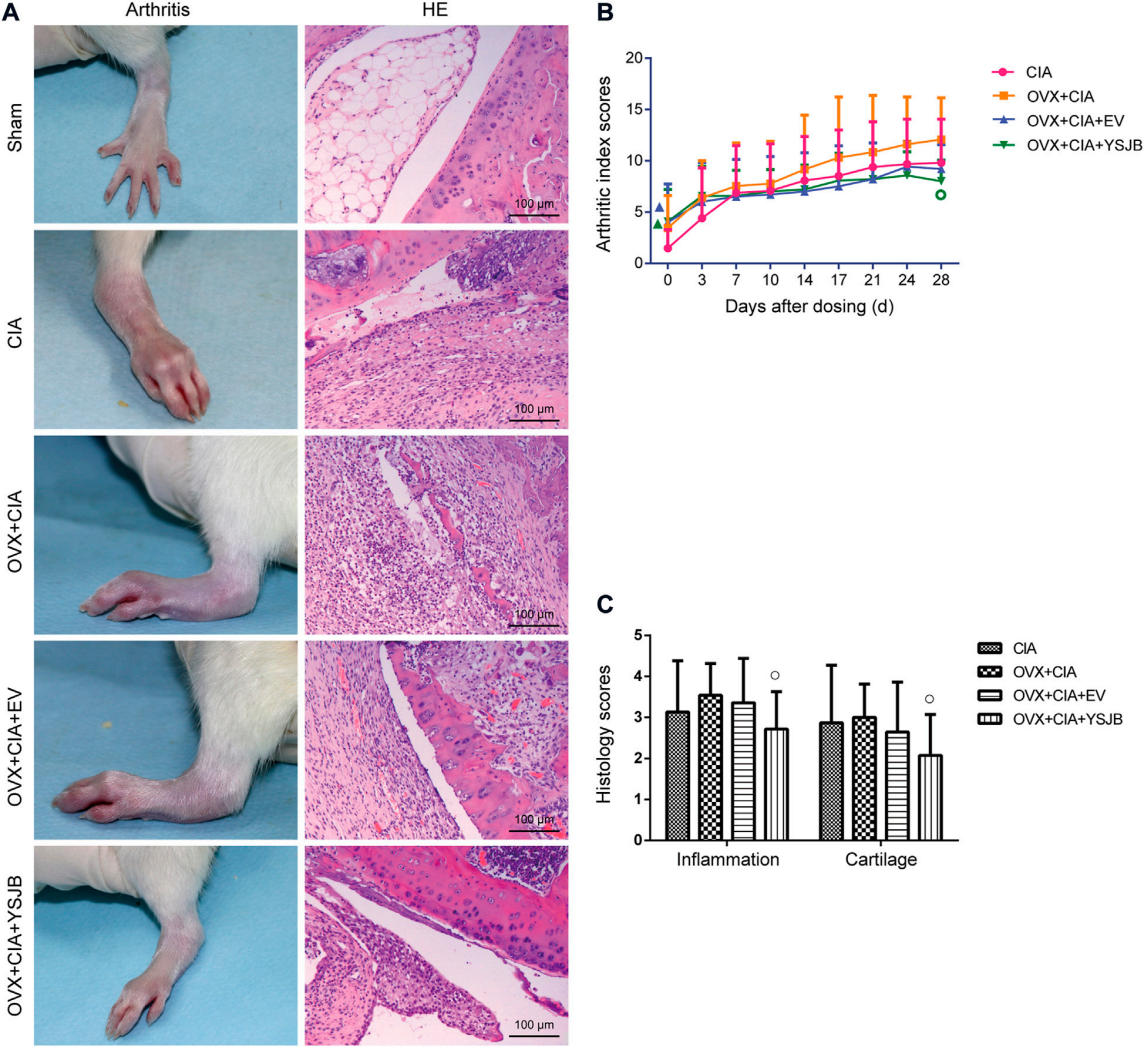
Effects of YSJB on arthritis progression in OVX + CIA rats. **(A)** Clinical signs of arthritis in hind paws, and representative pathological sections of the ankle and knee joints by H and E staining. **(B)** Line plots represent the arthritis index score. **(C)** Bar plots represent the inflammation score. Data are the mean ± *SD.* ^▲^ *p* < 0.05 compared with the CIA group. ^○^ *p* < 0.05 compared with the OVX + CIA group.

CIA induced inflammation and swelling in the hind paws of rats. The sham group did not show an increasing arthritis index scores for paws. Ovariectomy tended to enhance the mean arthritis index scores for paws and aggravate the clinical signs of arthritis in comparison with CIA induction in rats [Figure 1A (left)]. YSJB treatment lowered the arthritis index scores significantly compared with that in the OVX + CIA group [Figures 1A (left) and B].

Histology of the knee joints in the CIA group showed synovial hyperplasia, inflammatory-cell infiltration, cartilage damage, and bone erosion [Figure 1A (right)]. Ovariectomy tended to aggravate inflammatory-cell infiltration and damage to cartilage and bone compared with the CIA model (Figure 1C). We used semiquantitative grading scales to evaluate the influence of YSJB in inflamed joints: the histology scores were reduced significantly in comparison with those in the OVX + CIA group (*p* < 0.05) (Figure 1C).

### YSJB ameliorates bone destruction in OVX + CIA rats

**FIGURE 2:**
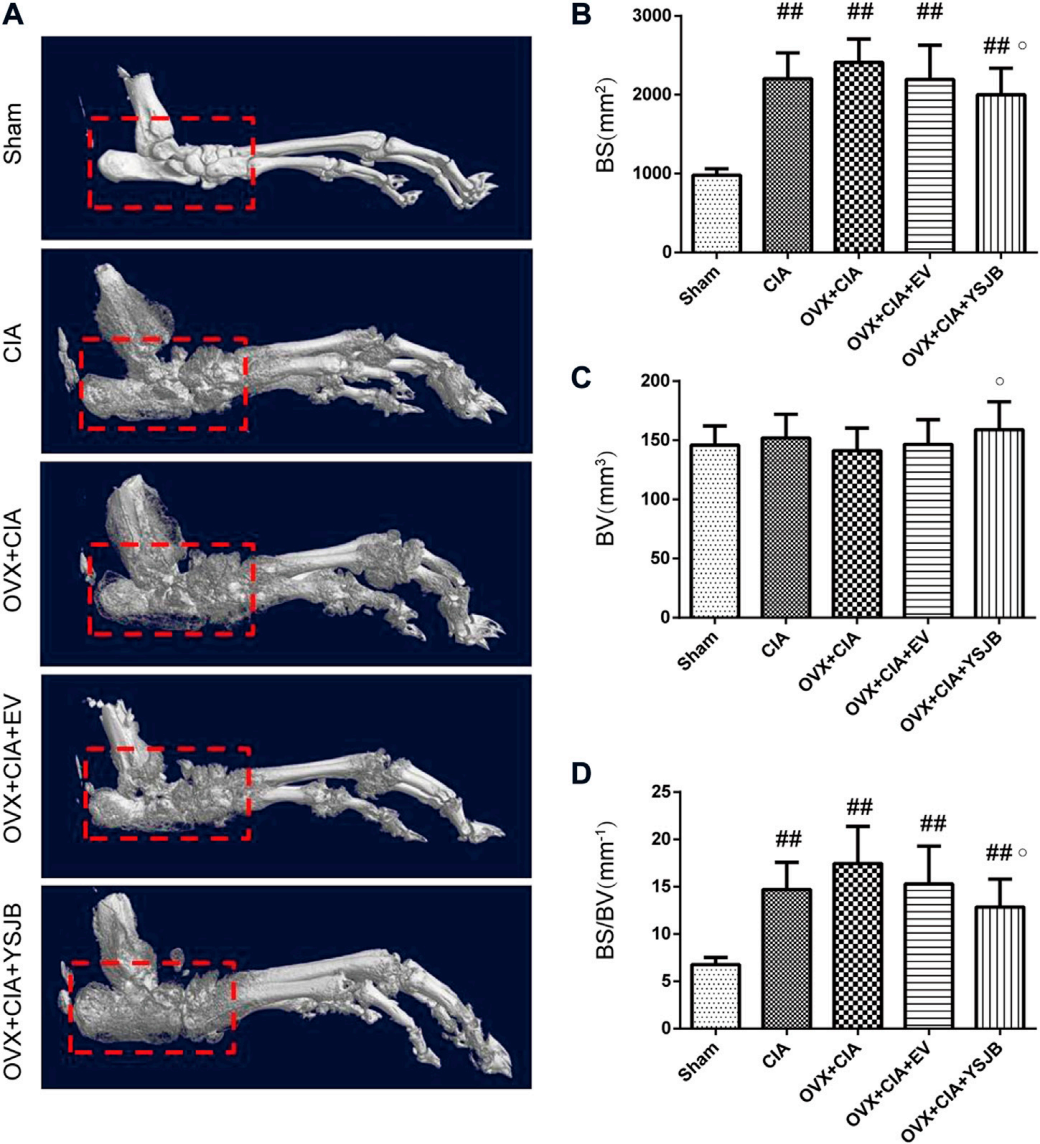
Micro-CT demonstrated. YSJB had a bone-protective effect on ankle joints. **(A)** Representative three-dimensional renditions of the ankle joint. **(B–D)** Bar plots of the bone surface, bone volume, and bone surface: bone volume ratio (BS/BV). Data are the mean ± *SD.* ^#^ *p* < 0.05, ^##^ *p* < 0.01 compared with the sham group. ^○^ *p* < 0.05 compared with the OVX + CIA group.

3D reconstructions based on micro-CT revealed the bone parameters of the hind paws and ankle joints. CIA induced bone destruction as quantified by BS (mm^2^), BV (mm^3^), and the BS/BV ratio (mm^−1^). The CIA, OVX + CIA, OVX + CIA + EV, and OVX + CIA + YSJB groups presented more severe bone damage than that in the sham group (Figure 2A), with significantly higher BS and the BS/BV ratio. The OVX + CIA + YSJB group had a markedly greater BV, but a reduction in BS and the BS/BV ratio, than those in the OVX + CIA group (Figure 2).

### YSJB reduced osteoclast differentiation in inflamed joints

**FIGURE 3:**
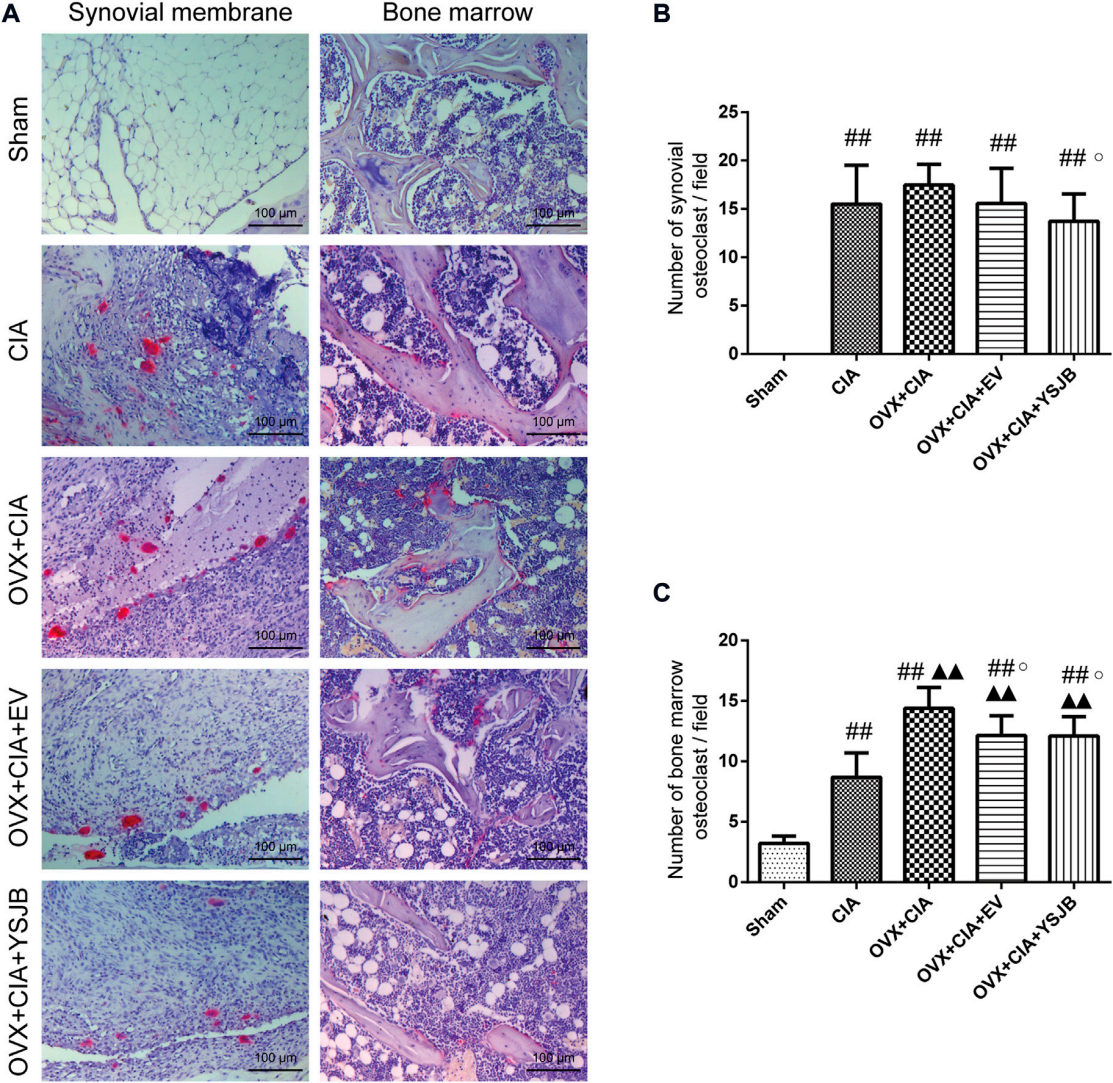
YSJB suppresses osteoclast differentiation in the synovial membrane and bone marrow of OVX + CIA rats. **(A)** Sections of ankle joints were stained with TRACP. **(B,C)** Evaluation of osteoclasts in the synovial membrane and bone marrow. Data are the mean ± *SD.* ^#^ *p* < 0.05, ^##^ *p* < 0.01 compared with the sham group. ^▲^ *p* < 0.05, ^▲▲^ *p* < 0.01 compared with the CIA group. ^○^ *p* < 0.05 compared with the OVX + CIA group.

Osteoclasts are the only cells involved in bone resorption. We used TRACP staining to observe osteoclast differentiation in the synovial membrane and BM (Figure 3A). TRACP^+^ osteoclasts were absent in the synovial membrane of rats in the sham group, but the BM contained a few osteoclasts. Compared with the sham group, CIA with/without OVX increased osteoclast differentiation significantly in the synovial membrane and BM. However, only in the BM, the OVX + CIA group showed significantly more osteoclasts than the CIA group. Moreover, YSJB treatment reduced the number of osteoclasts significantly in both regions, and EV treatment reduced only the number of osteoclasts in the BM (Figures 3B,C).

### Effects of YSJB on regulating the protein expression of ephrinB2, ephB4 and NFATc1 in the synovial membrane

**FIGURE 4:**
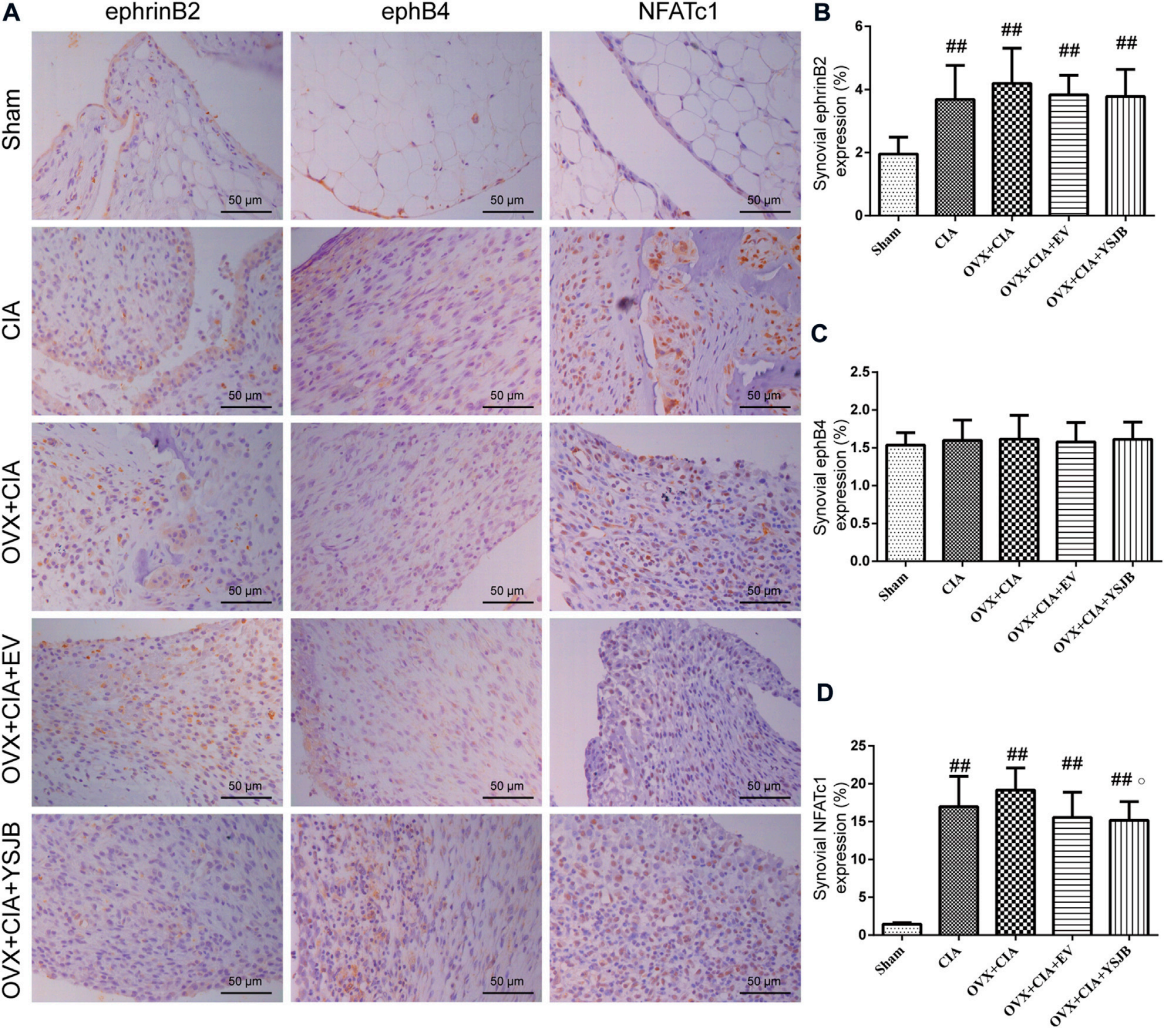
Effects of YSJB on regulation of the ephrinB2/ephB4/NFATc1 signaling pathway in the synovial membrane. Protein expression in paw sections was measured by immunohistochemistry. **(A)** Immunohistochemical staining showing protein expression of ephrinB2, ephB4, and NFATc1. **(B–D)** Bar plots represent mean protein expression of ephrinB2, ephB4, and NFATc1. Data are the mean ± *SD.* ^##^ *p* < 0.01 compared with the sham group. ^○^ *p* < 0.05 compared with the OVX + CIA group.

We wished to ascertain if ephrinB2–ephB4 signaling was involved in the inhibitory effects of YSJB upon osteoclast differentiation. We measured the expression of ephrinB2 and ephB4 as well as one osteoclastic transcription factor (NFATc1) in the synovium. Protein expression of ephrinB2, ephB4, and NFATc1 was measured in the synovial membrane of the joints by immunohistochemistry (Figure 4A). In the CIA, OVX + CIA, OVX + CIA + EV, and OVX + CIA + YSJB groups, the percentage of cells expressing ephrinB2 and NFATc1 was increased significantly (Figures 4B,D). In the synovial membrane, significant expression of ephrinB2 or NFATc1 was not observed in the EV group, but YSJB reduced NFATc1 expression significantly compared with that in the OVX + CIA group. No significant differences were found in ephB4 expression among groups (Figure 4C).

### YSJB regulates protein expression of ephrinB2 and NFATc1 in the BM

**FIGURE 5:**
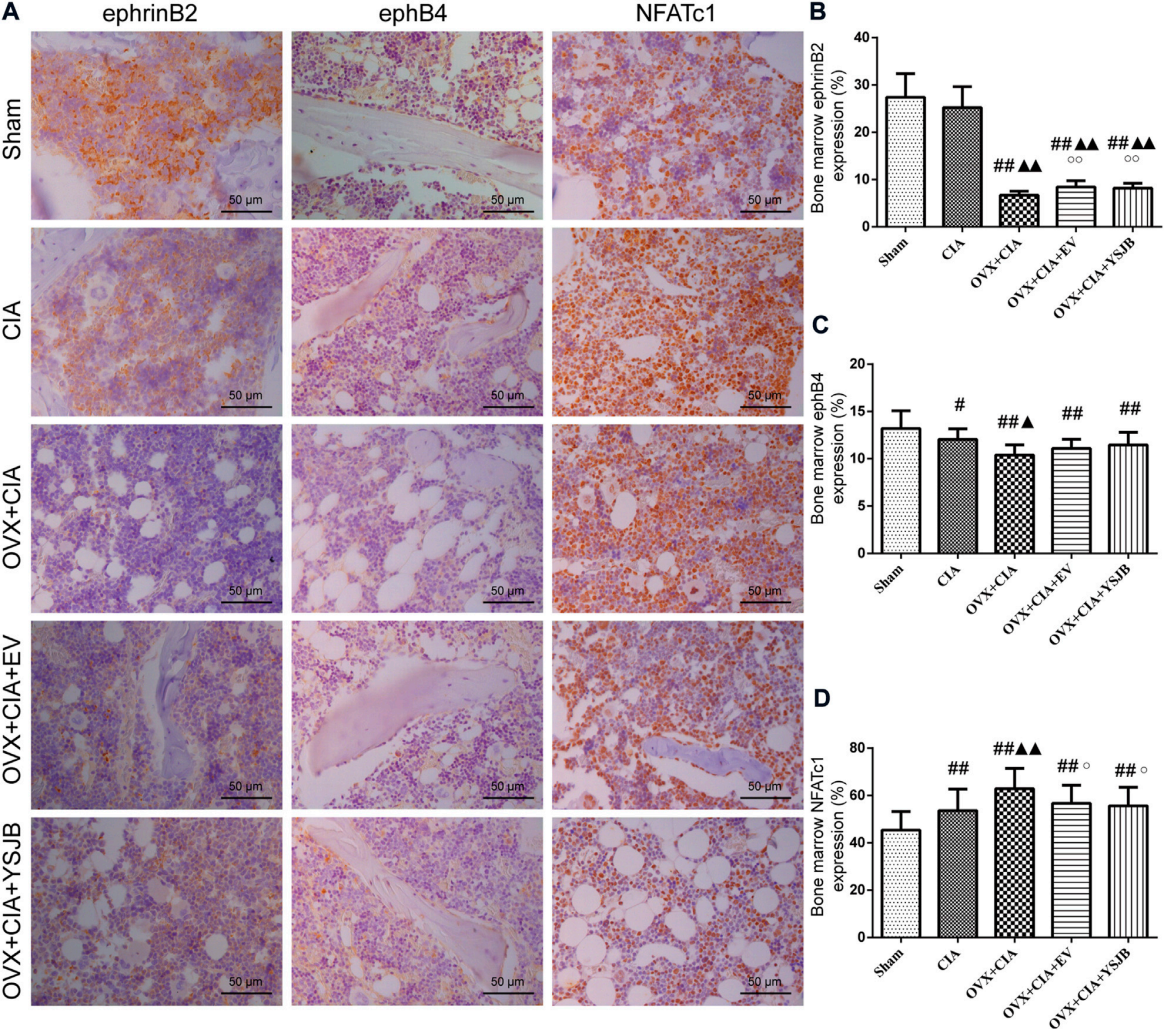
YSJB modulates the ephrinB2/ephB4/NFATc1 signaling pathway in the bone marrow to prevent bone destruction in OVX + CIA rats. **(A)** Immunohistochemical staining showing protein expression of ephrinB2, ephB4, and NFATc1 in the bone marrow. **(B–D)** Bar plots represent mean protein expression of ephrinB2, ephB4, and NFATc1. Data are the mean ± *SD.* ^#^ *p <* 0.05, ^##^ *p* < 0.01 compared with the sham group. ^▲^ *p* < 0.05, ^▲▲^ *p* < 0.01 compared with the CIA group. ^○^ *p* < 0.05 compared with the OVX + CIA group.

Based on the *in vivo* effects of YSJB upon osteoclast formation, we evaluated the effect of YSJB on regulation of the local protein expression of ephrinB2, ephB4, and NFATc1 in the BM (Figure 5A). In comparison with the sham group, the other four groups showed significantly downregulated ephB4 expression and upregulated NFATc1 expression in the BM. The OVX + CIA group, OVX + CIA + EV group, and OVX + CIA + YSJB group had reduced ephrinB2 expression compared with that in the sham group (Figure 5B). The OVX + CIA + YSJB group, OVX + CIA + EV group, and OVX + CIA group had significantly downregulated expression of ephrinB2 compared with that in the CIA group. The OVX + CIA group showed lower ephB4 expression and higher NFATc1 expression than that in the CIA group in the BM. YSJB treatment and EV treatment increased ephrinB2 expression markedly, but also reduced NFATc1 expression significantly (Figures 5B,D). However, no significant differences were observed in ephB4 expression in the BM upon EV treatment or YSJB treatment when compared with that in the OVX + CIA group (Figure 5C).

## Discussion

We explored the protective effects of YSJB upon articular microstructure by inhibiting osteoclast differentiation in CIA rats under postmenopausal conditions. YSJB modulated protein expression of ephrinB2, ephB4, and NFATc1 in the synovial membrane and BM of OVX+CIA rats. Post-menopausal status in RA is related to considerable damage and disability, and early menopause is a risk factor for RA (Sammaritano, 2012). Menopause is associated with worsening progression of functional decline. Women with RA during menopause had a worse disability of function compared with women with RA before the menopause (Mollard et al., 2018).

Inflammatory arthritis in postmenopausal women may be a complex outcome associated with estrogen deficiency and the immune system (Sapir-Koren and Livshits, 2017). Epidemiological studies have suggested that RA incidence is increased in postmenopausal women, which may be associated with estrogen deficiency. Stojanovic et al. showed that postmenopausal women with RA had a prominent reduction in bone mineral density (Stojanovic et al., 2021). However, we did not observe significantly increased destruction of local bone in OVX + CIA rats compared with CIA rats. Also, significant changes in the histology score were not observed in comparison with the CIA group, which could be attributed to the age of rats (the age at RA onset can affect the clinical picture and disease severity) (Banas et al., 2016). Heidari and Heidari. (2014) concluded that BMD loss in postmenopausal-onset RA was not greater than that in premenopausal age-matched controls, which was possibly because the estrogen deficiency regulating immunologic reactions compensates for the negative effects of estrogen deprivation on bone mass in post-RA patients. The role of estrogen deficiency in RA progression has been explored in animal models in which OVX complicates CIA. A recent *in vivo* study indicated that ovariectomy upregulated the expression of inflammatory factors in CIA animals as well as aggravating erosion of trabecular bone (Ibáñez et al., 2011). Reduced estrogen levels during menopause can lead to the development of a proinflammatory pattern that leads to RA onset (Chakraborty et al., 2022).

Estrogen has been reported to delay arthritis progression and protect articular cartilage in rats suffering from arthritis (Engdahl et al., 2018; Li and Li, 2020; Hang et al., 2021), but hormone replacement may no longer be an option (Stubelius et al., 2011). EV has been used to treat and prevent osteoporosis in studies (Li et al., 2020; Petr, 2021). Early administration of EV can significantly inhibit expression of the markers of high bone turnover in surgically induced menopause in women (Vatrasresth et al., 2021). However, we showed that administration of EV (a sex hormone) elicited little protective effect upon RA progression.

YSJB has been shown to regulate inflammatory and immunomodulatory responses in experimental models of arthritis. Perera et al. (2010); Perera et al. (2011) reported that YSJB decreased prostaglandin levels and increased expression of the pro-apoptotic Bax in the synovium of adjuvant arthritis (AA) rats, and downregulated expression of tumor necrosis factor-α, interleukin (IL)-1β mRNA, and caspase-3 in synoviocytes Previously, we showed that YSJB ameliorated bone loss and bone destruction in CIA rats, and that YJSB had a protective effect on the kidney deficiency induced by androgen deficiency in CIA rats (Zhao et al., 2012; Zhao et al., 2018). Here, we further explored the influence of YSJB on bone destruction in OVX + CIA rats as a model of premenopausal arthritis. YSJB treatment also reduced the histology score and arthritis index score of OVX + CIA rats by alleviating inflammatory-cell infiltration, cartilage and bone damage, and joint swelling. Moreover, YSJB exhibited a bone-protective effect according to micro-CT. Erosion of periarticular bone and bone destruction contribute considerably to RA pathogenesis. In RA, cartilage destruction accompanied by bone erosion in inflamed joints is related to increasing osteoclastogenesis. Osteoclasts are large, multinucleated, bone-resorbing cells derived from monocyte/macrophage progenitor cells. They have key roles in the destruction and loss of bone. Their excessive resorption activities are involved in the bone destructive observed in RA (Nishioku et al., 2020).

We showed that upregulation of osteoclast formation leading to bone resorption could be enhanced by ovariectomy, and that osteoclasts were activated markedly after upregulation of the inflammatory response. Inflammation of synovial tissue is a common feature of RA: it causes pannus formation within a joint (leading to secondary articular cartilage and bone erosion) and results in irreversible joint damage and disability. Local inflammation is considered to increase osteoclast activity, which results in local bone loss (Prieto-Potin et al., 2015). Luukkonen et al. found that the proinflammatory stimulus of synovial fluid during RA drives monocyte differentiation towards osteoclastogenesis *in vitro* (Luukkonen et al., 2022). Perera et al. (2010); Perera et al. (2011) reported that YSJB decreased prostaglandin levels and increased expression of the pro-apoptotic Bax in the synovium of AA rats, and downregulated expression of TNF-α, IL-1β mRNA, and caspase-3 in synoviocytes. We observed osteoclast differentiation in synovial tissue. YSJB treatment to restore the upregulation of osteoclast numbers and expression of the essential osteoclastic transcription factor NFATc1 in the synovial membrane and BM of OVX + CIA group.

EphrinB2–ephB4 signaling has important regulatory roles in skeletal homeostasis *via* communication between osteoclasts and osteoblasts; ephrinB2 has a negative impact upon osteoclasts, whereas ephB4 has a positive effect on osteoblasts (Zhao et al., 2006). EphrinB2 ligand is expressed simultaneously on the osteoclast membranes and osteoblast membranes. It can be activated by the ephB4 receptor, which is expressed on the osteoblast membrane to inhibit osteoclast differentiation (Ge et al., 2020). Several studies have shown that ephrinB2 and ephB4 participate in various bone-related diseases (Shen et al., 2016; Valverde-Franco et al., 2016; Wu et al., 2016).

However, YSJB did not show a significantly altered effect on expression of ephrinB2 and ephB4 in synovial tissue. Kurowska et al. suggested that the BM should be taken into consideration when studying therapeutic interventions aimed at osteoclast activation in bone resorption in RA (Kurowska et al., 2021). Hence, we also, observed regulation of expression of ephrinB2 and ephB4 in the BM. Consistent with the findings of Kurowska et al. targeting osteoclasts in the BM may be efficacious treatment for postmenopausal inflammatory arthritis.

Variations in estrogen levels lead to differential regulation of protein expression, and their related signaling is directly/indirectly associated with RA pathogenesis. We found that expression of ephrinB2 and ephB4 was markedly lower in the BM, and that YSJB increased ephrinB2 expression significantly (but not ephB4 expression) in the BM. Osteoclasts in the BM were negatively correlated with ephrinB2 expression. In addition, we noted decreased expression of ephrinB2 and ephB4, but increased osteoclast differentiation and NFATc1 expression in the BM of rats in the OVX + CIA group compared with the CIA group. Zhang et al. reported that enhancement of ephrinB2–ephB4 signaling could inhibit osteoclastogenesis and prevent bone loss in OVX rats, and that knockdown of ephrinB2 expression reversed the inhibitory effect upon osteoclasts (Zhang et al., 2022). Huang et al. (2020) showed that modulating the balance of the ephB4–ephrinB2 axis could improve the characteristics of osteoporosis. EphB4–ephrinB2 signaling affects the osteoclastic factors RANK ligand/osteoprotegerin (Li et al., 2021). Differential outcomes of ephB4–ephrinB2 signaling may offer formidable challenges for the development of RA therapeutics. YSJB upregulated ephrinB2 expression significantly in the BM and downregulated NFATc1 expression in rats of OVX + CIA group. In this model of postmenopausal arthritis, YSJB administration improved arthritis progression and prevented bone destruction by reducing osteoclastogenesis and regulating the expression of ephrinB2, ephB4, and NFATc1 in the BM. Our results suggest that modulation by YSJB of ephrinB2 signaling in the BM (but not the synovium) ameliorates bone destruction in CIA. Further studies may characterize the precise mechanisms underlying the involvement of the ephrinB2–ephB4 axis in the antiresorptive YSJB treatment of RA.

## Conclusion

YSJB exhibits a bone-protective effect and it may be a promising therapeutic strategy for alleviating bone destruction in arthritis under postmenopausal conditions. Moreover, one of the mechanisms is associated with the modulation of ephrinB2 signaling.

## Data Availability

The raw data supporting the conclusions of this article will be made available by the corresponding author, without undue reservation.
